# Supplementary material for: Self-assembly of multi-stranded perylene dye J-aggregates in columnar liquid-crystalline phases
Source: Nat Commun. 2018 Jul 6;9:2646. doi: 10.1038/s41467-018-05018-6 (PMC6035248; doi:10.1038/s41467-018-05018-6)
Supplement: Supplementary file 1 — Supplementary Information [file 41467_2018_5018_MOESM1_ESM.pdf]

Self-assembly of Multi-Stranded Perylene Dye J-Aggregates in Columnar Liquid Crystalline Phases

Herbst et al.

Supplementary Methods

Reagents were purchased from commercial suppliers (Sigma-Aldrich, ACROS, Alfa Aeser, TCI and Merck) and used as received without further purification with the exception of *N,N'*-dicyclohexylcarbodiimide (DCC), which was distilled in prior. All the organic solvents were distilled and dried by standard procedures. All reactions were carried out under nitrogen atmosphere. Column chromatography was performed with commercial glass columns using silica gel 60M (particle size 0.04-0.063 mm) from Macherey-Nagel as stationary phase.

NMR spectroscopy: ^{1}H and ^{13}C nuclear magnetic resonance (NMR) spectra were recorded on a Bruker-Daltonics *Avance-400* spectrometer operating at 400 MHz (^{1}H) or 100 MHz (^{13}C), with the residual protic solvent used as the internal standard. The chemical shifts are reported in parts per million (ppm). Multiplicities for proton signals are abbreviated as s, d, and m for singlet, doublet and multiplet, respectively.

Mass spectrometry: High resolution mass spectra (HRMS) were recorded on an ESI *micrOTOF focus* spectrometer (Bruker Daltonic GmbH, Germany).

Elemental analyses were performed on a CHN 932 analyzer (Leco Instruments GmbH, Mönchengladbach, Germany).

Polarizing optical microscopy (POM): Optical textures of the liquid crystalline materials were examined with a Nikon Eclipse *LV100Pol* optical polarizing microscope equipped with a Linkam LTS420 heating stage and a Linkam T95-HS system controller.

Differential Scanning Calorimetry (DSC): Thermal analyses by differential scanning calorimetry were performed on a TA instrument *DSC Q1000* with a DSC refrigerated cooling system.

Synthesis of precursors. Compound **5** was synthesized as described in the literature.[1] Compounds **7-9** were synthesized according to previously described procedures.[1,2,3] Pyridinium *p*-toluenesulfonate (DPTS) was synthesized following literature known procedure.[4] Syntheses of the compounds **7b**, **8b** are described in our previous paper.[2] Syntheses of the compounds **3** ,**7c**, **8c** and **9** are described in literature.[1,3]

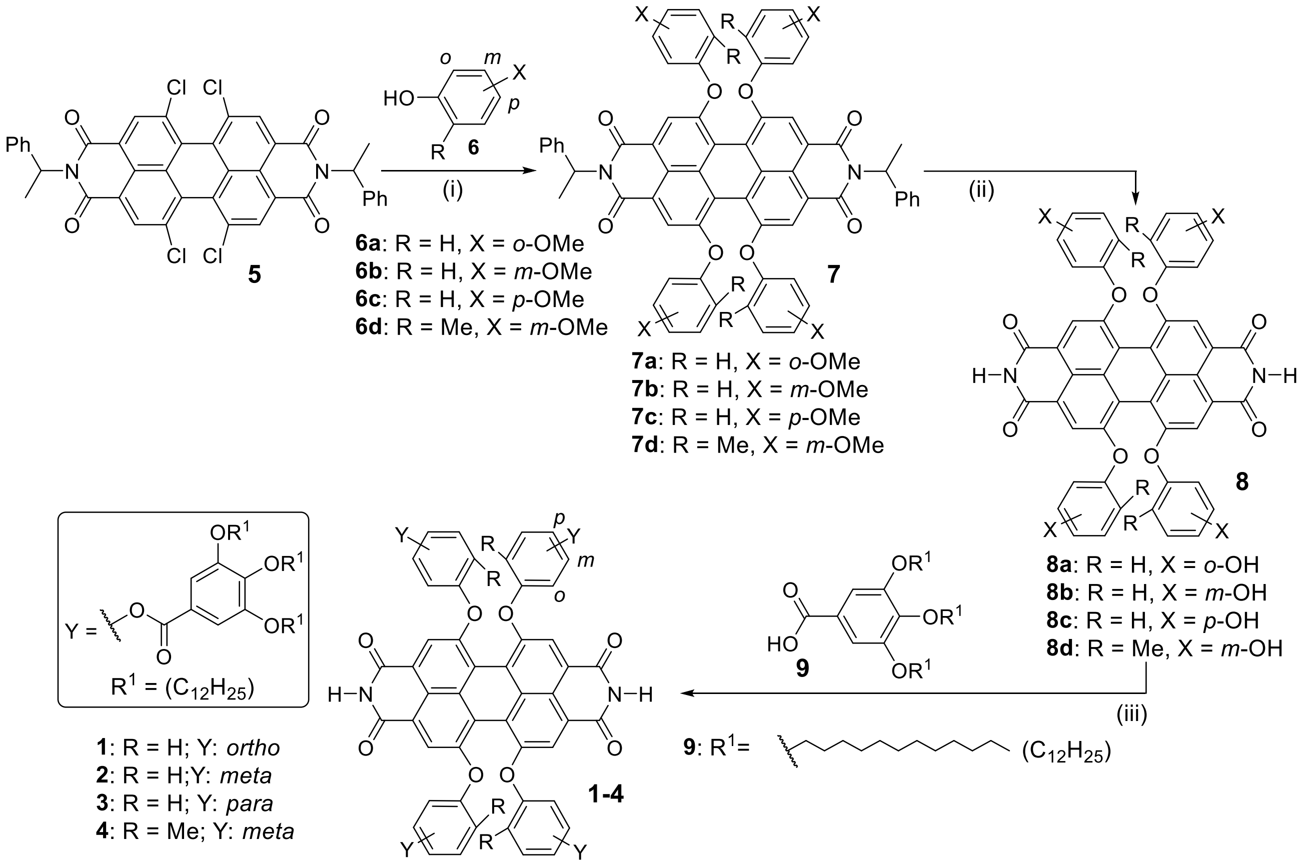

Supplementary Figure 1. **Synthetic procedure for tetra-bay substituted PBIs 1-4.** Reagents and conditions: (i) **6a-d**, K_2CO_3, DMF, 130 °C. (ii) BBr_3, CH_2Cl_2, 0 → 25 °C. (iii) **9**, DCC, DPTS, DMF/CH_2Cl_2, r.t.

General Procedure for the preparation of 7a-d. A suspension of **5**, the appropriate methoxyphenol (**6a-d**) and K_2CO_3 was stirred in anhydrous *N*-methyl-2-pyrrolidone (NMP) at 130 °C under argon atmosphere for 12 h. The reaction mixture was cooled down to room temperature and precipitated by addition of 1 N HCl until reaching pH 1. The mixture was stirred for 1 h before the filtration to collect the solid, followed by washing with water and MeOH. The crude solid was dried under vacuum and purified by column chromatography (SiO_2, CH_2Cl_2). The concentrated solution in dichloromethane was added into ice-cooled methanol to precipitate the final product as a dark red solid.

Compound 7a. **5** (1.00 g, 1.36 mmol), **6a** (0.84 g, 6.79 mmol) and K_2CO_3 (0.94 g, 6.79 mmol) in 20 mL of NMP. The reaction gave 1.03 g of **7a** in 70% yield.

^{1}H NMR (400 MHz, $CDCl_3$, ppm): δ = 7.99 (s, 4H), 7.40 (d, ^3J = 7.6 Hz, 4H), 7.27 (m, 4H), 7.19 (m, 4H), 7.08 (m, 4H), 7.00 (m, 4H), 6.89 (m, 4H), 6.79 (m, 4H), 6.44 (m, 2H), 3.60 (s, 12H), 1.90 (d, ^3J = 6.8 Hz, 6H). ^{13}C NMR (100 MHz, $CDCl_3$, ppm) δ = 163.7, 156.2, 151.4, 143.3, 140.9, 133.1, 128.3, 127.0, 126.9, 125.9, 122.5, 122.3, 121.3, 119.8, 119.4, 118.5, 112.6, 55.7, 50.1, 16.2. HRMS (ESI, acetonitrile/chloroform 1:1, pos. mode): *m/z* calculated for $C_{68}H_{50}N_2NaO_{12}$: 1109.3260 $[M+Na]^+$, found: 1109.3257.

Compound 7d. 5 (1.00 g, 1.36 mmol), **6d** (0.94 g, 6.79 mmol) and K_2CO_3 (0.94 g, 6.79 mmol) in 40 mL anhydrous NMP. The reaction gave 0.67 g of **7b** in 43% yield.

^{1}H NMR (400 MHz, $CDCl_3$, ppm): δ = 8.01 (s, 4H), 7.43 (d, ^3J = 7.6 Hz, 4H), 7.29 (m, 4H), 7.21 (m, 4H), 7.08 (d, ^3J = 8.6 Hz, 4H), 6.63 (dd, J = 2.6, 8.4 Hz, 4H), 6.47 (m, 6H), 3.49 (s, 12H), 1.98 (s, 12H), 1.91 (d, ^3J = 7.1 Hz, 6H). ^{13}C NMR (100 MHz, $CDCl_3$, ppm) δ = 163.6, 159.2, 156.3, 153.5, 140.7, 133.2, 131.9, 128.3, 127.1, 123.0, 122.0, 119.8, 119.4, 118.8, 112.0, 106.6, 55.3, 50.3, 16.2, 15.4. HRMS (ESI, acetonitrile/chloroform 1:1, pos. mode): *m/z* calculated for $C_{72}H_{58}N_2NaO_{12}$: 1165.3882 $[M+Na]^+$, found: 1165.3866.

General Procedure for the preparation of 8a-d. Into a solution of **7a-d** in dry CH_2Cl_2 was added a solution of BBr_3 in dry CH_2Cl_2 (40 mL) dropwise at 0 °C under argon atmosphere. The reaction mixture was stirred at 0 °C for 1 h and further 3.5 h at 25 °C. The solvent and excess of BBr_3 was removed by distillation and 50 mL of MeOH/water (1:4) mixture was added slowly to quench the reaction. The resultant suspension was sonicated for 30 min. The solid was collected by filtration and dried under vacuum to give a dark blue solid in quantitative yield. The solid was used for the next step without further purification.

Compound 8a. 7a (0.80 g, 0.74 mmol) in CH_2Cl_2 (70 mL) and BBr_3 (3.69 g, 14.7 mmol) in dry CH_2Cl_2 (40 mL). The reaction gave 0.61 g of crude **8a**.

^{1}H NMR (400 MHz, $(CD_3)_2CO$, ppm): δ = 11.85 (s, 2H), 9.58 (s, 4H), 7.71 (s, 4H), 7.10 (m, 4H), 7.00 (m, 8H), 6.80 (m, 4H). ^{13}C NMR (100 MHz, $(CD_3)_2CO$, ppm) δ = 163.6, 155.7, 149.3, 141.4, 132.7, 126.6, 122.6, 122.4, 120.1, 119.3, 118.2, 117.5, 115.9. MS (MALDI-TOF, neg. mode, DCTB): *m/z* calculated for $C_{48}H_{25}N_2O_{12}$ 821.14 $[M-H]^-$; found 821.12.

Compound 8d. 7d (0.95 g, 0.83 mmol) in dry CH_2Cl_2 (80 mL) and BBr_3 (4.16 g, 16.6 mmol) in dry CH_2Cl_2 (40 mL) was reacted to give 0.73 g of crude **8d**.

^{1}H NMR (400 MHz, $(CD_3)_2CO$, ppm): δ = 11.98 (s, 2H), 7.79 (s, 4H), 7.16 (d, ^{2}J = 8.4 Hz, 4H), 6.63 (dd, J = 8.3, 2.4 Hz, 4H), 6.45 (d, ^{4}J = 2.4 Hz, 4H), 1.98 (s, 12H). ^{13}C NMR (100 MHz, $(CD_3)_2CO$, ppm) δ = 163.5, 156.8, 155.6, 153.3, 132.9, 131.9, 123.1, 119.7, 119.6, 118.5, 116.8, 112.7, 107.6, 14.9. MS (MALDI-TOF, neg. mode, DCTB): *m/z* calculated for $C_{52}H_{33}N_2O_{12}$ 877.20 $[M-H]^-$; found 877.21.

PBI 1. Into the solution of **8a** (130 mg, 0.16 mmol), **9** (500 mg, 0.70 mmol) and DPTS (90.0 mg, 0.32 mmol) in freshly distilled DMF (0.5 mL) and CH_2Cl_2 (1.5 mL) were added 4-5 beads molecular sieves (4 Å) and a solution of DCC (170 mg, 0.82 mmol) in CH_2Cl_2 (0.5 mL) in one portion under argon atmosphere. The reaction mixture was stirred at room temperature for 12 h under argon atmosphere. The solution was concentrated in vacuum and the solid residue was purified by column chromatography (SiO_2, CH_2Cl_2) followed by the precipitation in cold MeOH and drying under vacuum (50 °C, 10^{-2}-10^{-3} mbar) to give 350 mg (64 %) of a red solid.

^{1}H NMR (400 MHz, $CDCl_3$, ppm): δ = 8.18 (s, 2H), 8.13 (s, 4H), 7.12 (m, 16H), 6.74 (s, 8H), 3.73 (br. s, 8H), 3.45 (br. s, 16H), 1.63-1.10 (m, 240H), 0.79 (m, 36H). ^{13}C NMR (100 MHz, $CDCl_3$) δ = 163.6, 162.2, 155.3, 152.4, 147.3, 147.1, 142.7, 141.4, 132.7, 127.1, 125.0, 124.2,122.7, 122.2, 120.6, 120.4, 120.0, 107.6, 73.4, 68.8, 32.1, 30.6, 30.0, 29.9, 29.9, 29.6, 29.6, 29.4, 26.3, 26.1, 22.9, 14.3. HRMS (ESI, acetonitrile/chloroform 1:1, pos. mode): *m/z* calculated for $C_{220}H_{331}N_2O_{28}$: 3449.4533 $[M+H]^+$, found: 3449.4525. Elemental analysis (%) calculated for $C_{220}H_{330}N_2O_{28}$ (3450.97): C 76.57, H 9.64, N 0.81; found: C 76.86, H 9.84, N 0.87.

PBI 2. Into the solution of **8b** (65.0 mg, 79.0 µmol), **9** (256 mg, 395 µmol) and DPTS (45.0 mg, 158 µmol) in freshly distilled DMF (0.5 mL) and CH_2Cl_2 (0.5 mL) were added 2-3 beads molecular sieves (4 Å) and a solution of DCC (100 mg, 466 µmol) in CH_2Cl_2 (0.5 mL) in one portion under argon atmosphere. The reaction mixture was stirred at room temperature for 5 d under argon atmosphere. The solution was concentrated in vacuum and the solid residue was purified by column chromatography (SiO_2, $CHCl_3$/hexane (40 to 20 to 10 %)) followed by the precipitation in cold MeOH and drying under vacuum (50 °C, 10^{-2}-10^{-3} mbar) to give 158 mg (58 %) of a dark blue solid.

^{1}H NMR (400 MHz, $CDCl_3$, ppm): δ = 8.38 (s, 2H), 8.28 (s, 4H), 7.31 (m, 12H), 6.97 (d, 4H, J = 7.9 Hz), 6.89 (d, 4H, J = 8.8 Hz), 6.84 (t, 4H, J = 1.9 Hz), 4.04-3.95 (m, 24H), 1.82-1.71 (m, 24H), 1.52-1.41 (m, 30H), 1.26 (br s, H), 0.89-0.85 (m, 36H). ^{13}C NMR (100 MHz, $CDCl_3$) δ = 164.4, 162.6, 155.9, 155.2, 152.9, 152.2, 143.1, 130.5, 123.5, 122.9, 121.0, 118.2, 116.9, 113.8, 108.5, 73.6, 69.2, 31.9, 30.4, m 29.8, 29.7, 29.6, 29.5, 29.4, 29.3, 26.1, 26.1, 22.7, 14.1. HRMS (ESI, acetonitrile/chloroform 1:1, pos. mode): *m/z* calculated for $C_{220}H_{331}N_2O_{28}$ 3449.4553 $[M+H]^+$, found 3449.4533 (Δ 0.76 ppm). Elemental analysis (%) calculated for $C_{220}H_{330}N_2O_{28}$ (3450.97): C 76.57, H 9.64, N 0.81; found: C 76.52, H 9.89, N 0.78.

PBI 4. Into the solution of **8d** (140 mg, 0.16 mmol), **9** (470 mg, 0.70 mmol) and DPTS (90.0 mg, 0.32 mmol) in freshly distilled DMF (0.5 mL) and CH_2Cl_2 (1.5 mL) were added 4-5 beads molecular sieves (4 Å) and a solution of DCC (170 mg, 0.82 mmol) in CH_2Cl_2 (0.5 mL) in one portion under argon atmosphere. The reaction mixture was stirred at room temperature for 24 h under argon atmosphere. The solution was concentrated in vacuum and the solid residue was purified by column chromatography (SiO_2, CH_2Cl_2/Et_2O (0 to 3 %)) followed by precipitation in cold MeOH and drying under vacuum (50 °C, 10^{-2}-10^{-3} mbar) to give a dark blue solid: 340 mg (62%).

^{1}H NMR (400 MHz, Chloroform-*d*) δ 8.38 (s, 2H), 8.15 (s, 4H), 7.30 (s, 8H), 6.93 (dd, *J* = 8.3, 2.4 Hz, 4H), 6.86 (d, *J* = 2.4 Hz, 4H), 4.00 (dt, *J* = 10.8, 6.5 Hz, 24H), 2.08 (s, 12H), 1.85 – 1.69 (m, 25H), 1.50 – 1.41 (m, 24H), 1.37 – 1.23 (m, 192H), 0.92 – 0.84 (m, 36H). ^{13}C NMR (100 MHz, $CDCl_3$) δ = 164.3, 162.9, 156.1, 153.5, 153.0, 150.1, 143.0, 133.6, 132.1, 127.2, 123.9, 122.7, 120.9, 120.6, 119.4, 118.8, 113.9, 108.5, 73.7, 69.3, 32.1, 30.5, 29.9, 29.8, 29.7, 29.6, 29.5, 26.3, 26.2, 22.8, 15.8, 14.3. HRMS (ESI, acetonitrile/chloroform 1:1, pos. mode): *m/z* calculated for $C_{224}H_{338}N_2NaO_{28}$: 3527.49784 $[M+Na]^+$, found: 3527.49454. Elemental analysis (%) calculated for $C_{224}H_{338}N_2O_{28}$ (3507.15): C 76.71, H 9.71, N 0.80; found: C 77.32, H 9.75, N 0.89.

Density measurements by the buoyancy method at 20 °C

Density measurements were carried out in mixtures of deionized water and aqueous sodium chloride (20 wt%) solution. Prior to dissolving, sodium chloride (*pro analysi*) was dried at 150 °C under reduced pressure (1×10^{-3} mbar). Both solvents were degassed by ultrasonication. The samples were molten to the isotropic liquid in order to avoid inclusion of air. Subsequently, the samples were extruded into a thin solid fiber and cut into a number of small pieces of varying size (0.1 – 0.6 mg). The samples were put in a sealed vial containing deionized water at 20 °C. Aqueous sodium chloride (20 wt%) solution was added in small portions until the sample started floating. The mixture was allowed to equilibrate between additions. The necessary weight percentage of sodium chloride was determined and the density was calculated according to reference 5.

Results:

PBI 2: $\delta = 1.018 \pm 0.006$ g cm^{-3}.

PBI 4: $\delta = 1.028 \pm 0.009$ g cm^{-3}.

Note that this method relies on samples which are free from air inclusions, which cannot be guaranteed strictly with the present preparation of the samples. For materials with much lower clearing temperatures and high thermal stability in the isotropic liquid, the samples can be prepared by keeping them a long time in the isotropic liquid under vacuum, which is supposed to eliminate all air bubbles.[S6] However, in the present case this is not possible since the PBIs decompose slowly at such high temperatures (>240 °C). Therefore, the present density value is a lower limit for **PBI 2** and **PBI 4**.

Extrapolation of the molecular volumes at higher temperatures and the calculation of the numbers of molecules per columnar stratum (PBI 2, PBI 3 and PBI 4)

From the density measurements at 20 °C the molecular volumes can be obtained by $V_{mol} = \delta/N_A/M$ (δ = density, N_A = Avogadro's constant, M molecular mass). The temperature dependence of the molecular volume of a perylene bisimide with branched chains was reported previously in reference 7. Assuming the known temperature-dependence of the volume of aliphatic chains (CH, CH_2, CH_3) from references 8, 9 the volume gradient of the aromatic unit was calculated to be 0.0174 Å^3/°C.

Assuming that this volume gradient is close to the volume gradient within the present perylene bisimide scaffolds, the molecular volumes V_{mol} based on the experimental values of PBI **2** and PBI **4**, the volumes of the aliphatic chains V_{CH} and the aromatic units $V_{ar} = V_{mol}$-V_{CH} were calculated at 20 °C.[S8] With the known temperature-dependence of V_{CH} and the calculated volume change for the aromatic unit the molecular volume could be calculated with $V_{mol}(T) = V_{CH}(T) + V_{ar}(T)$. These values are summarized in Supplementary Table 1.

Supplementary Table 1. Molecular volumes, densities and number of molecules per columnar stratum.

N°	T / °C	V_{CH} / Å^3	V_{ar} / Å^3	V_{mol} / Å^3	δ / g*cm^{-3}	axial separation of subunits / Å	Number of molecules per columnar stratum
PBI **2**	20	1737.9	3891.3	5629.2	1.018[a]	-	-
PBI **2** (Col$_r$)	160	1740.3	4420.4	6160.7	0.930	13.8	3.7 ± 0.3
PBI **2** (Col$_h$)	200	1741.0	4607.7	6348.7	0.903	13.8	3.0 ± 0.2
PBI **3**[b] (Col$_h$)	180	1740.6	4512.0	6252.7	0.916	14.1	3.2 ± 0.2
PBI **4**	20	1773.8	3891.3	5665.1	1.028[a]	-	-
PBI **4** (Col$_h$)	224	1777.4	4727.8	6505.1	0.895	14.2	2.0 ± 0.1

[a] experimental values obtained by the buoyancy method at 20 °C. [b] Values obtained assuming the same molecular volume than PBI **2** at 20 °C, owing to the identical molecular composition.

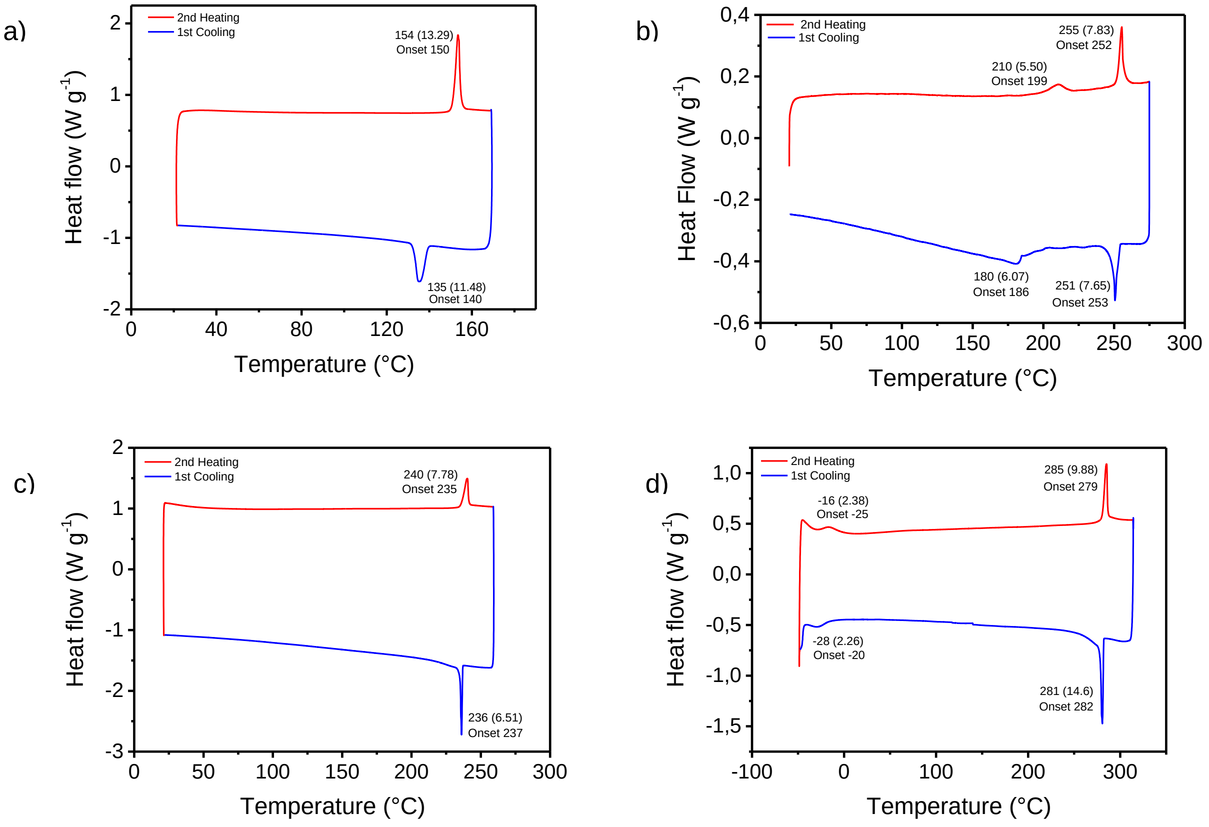

Supplementary Figure 2. Differential scanning calorimetry (DSC) of PBIs 1-4. Differential scanning calorimetry (DSC) traces of a) PBI **1**, b) PBI **2**, c) PBI **3** and d) PBI **4** in the second heating and first cooling cycles. The heating/cooling rate was 10 °C/min. The numbers stated close to the transitions indicate the melting temperatures in °C and the numbers in parenthesis correspond to the transition enthalpies Δ*H* in kJ/mol.

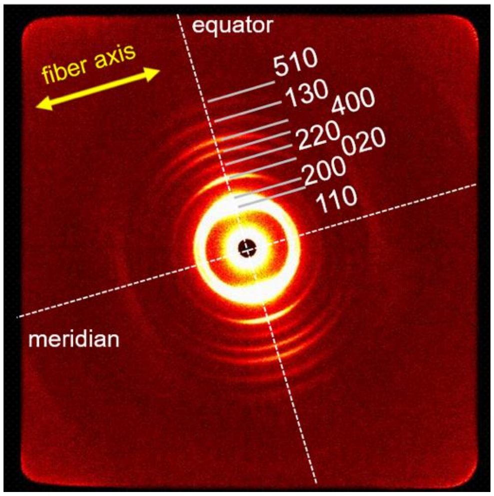

Supplementary Figure 3. MAXS pattern of PBI 2. MAXS pattern of an extruded fiber of PBI **2** at 160 °C.

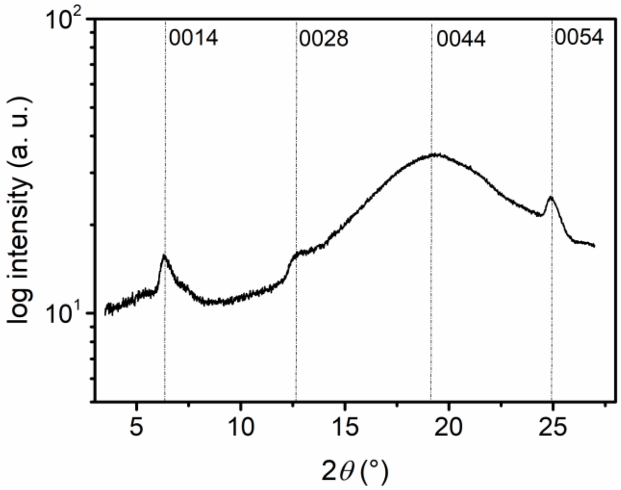

Supplementary Figure 4. Integrated intensities of the WAXS pattern of PBI 2. Integrated intensities along the meridian (right) of the WAXS pattern of an aligned fiber of PBI **2** (160 °C) with the position of the meridional reflections 0014, 0028, 0044 and 0054 corresponding to the helical self-assembly.

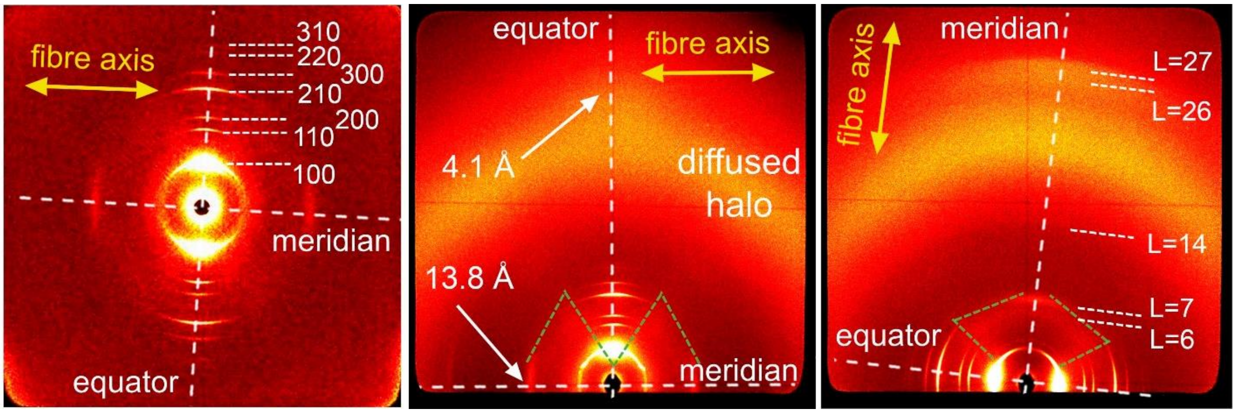

Supplementary Figure 5. MAXS and WAXS patterns of PBI 2. MAXS (left) and WAXS (middle and right) patterns of **2** at 200 °C of a lying (left and middle) and a standing fiber (right). The positions of the equator and meridian are indicated with white dashed lines. Direction of the fiber is indicated by yellow arrows.

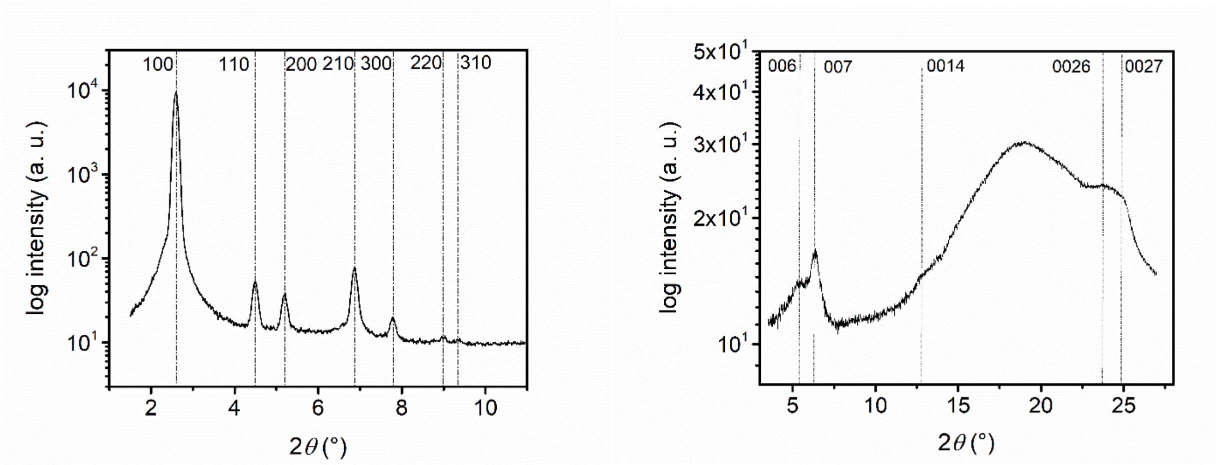

Supplementary Figure 6. Integrated intensities of the MAXS pattern of PBI 2. Integrated intensities along the equator (left) of the MAXS pattern of PBI **2** at 200 °C, showing the reflections indexed according to the Col_h phase p6mm (a = 39.9 Å). Integrated intensity along the meridian (right) of the WAXS pattern of PBI **2** at 200 °C with the position of the meridional reflections 006, 007, 0014, 0026 and 0027 corresponding to the helical self-assembly.

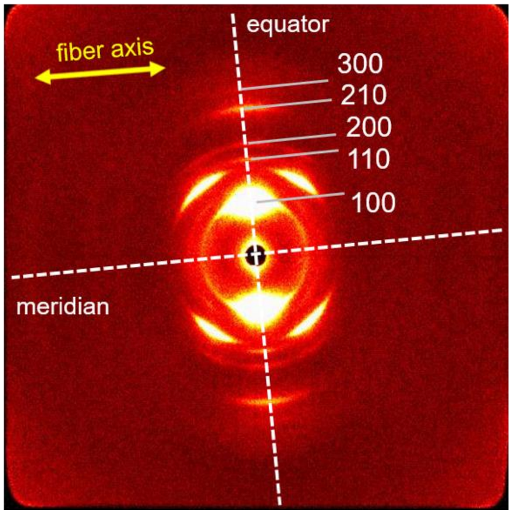

Supplementary Figure 7. MAXS pattern of PBI 3. MAXS pattern of an extruded fiber of PBI **3** at 180°C.

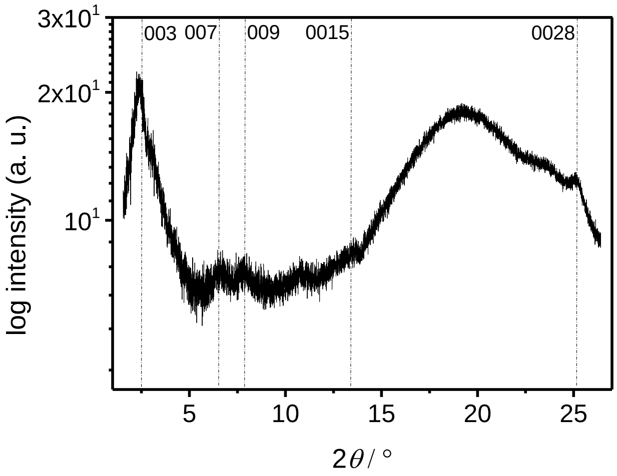

Supplementary Figure 8. Integrated intensities of the WAXS pattern of PBI 3. Integrated intensity along the meridian (right) of the WAXS pattern of **3** at 180 °C. In the pattern the positions of possible meridional signals *00l* are given as an orientation. Only the meridional reflections 007, 009 and 0028 are found on these lines, the other diffuse signals are off-meridional reflections positioned at the corresponding layer lines on both sides of the meridian.

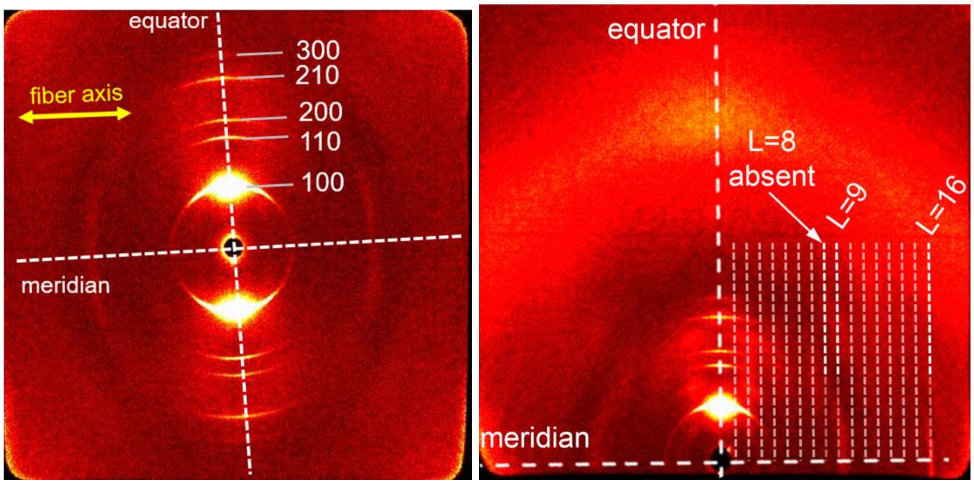

Supplementary Figure 9. MAXS pattern of PBI 4. (Left) MAXS pattern of an extruded fiber of PBI **4** at 224 °C. (Right) WAXS pattern of a lying fiber of PBI **4** at 163 °C with clear diffuse signals at small angles. Reflection on L = 8 is absent. A strong meridional reflection is observed at layer line L = 16.

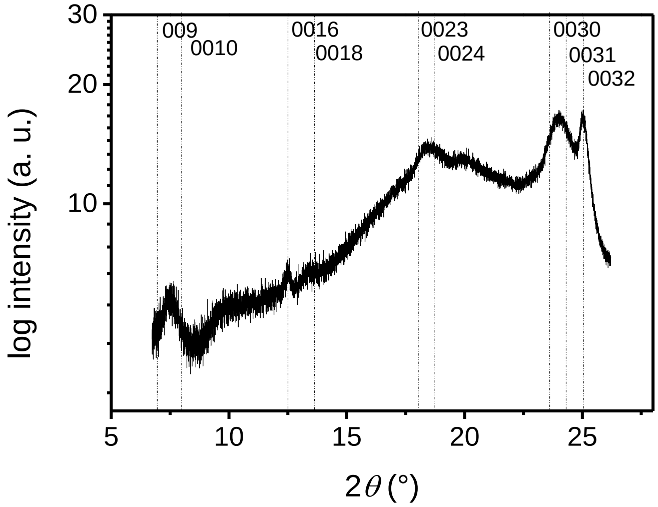

Supplementary Figure 10. Integrated intensities of the WAXS pattern of PBI 4. Integrated intensity along the meridian of the WAXS pattern of **4** at 224 °C. In the pattern the positions of possible meridional signals 00l are given as an orientation. Only the meridional reflections 0016, 0018 and 0032 are found on these lines, the other diffuse signals are off-meridional reflections positioned at the corresponding layer lines on both sides of the meridian.

Supplementary Note 1. Middle and Wide Angle X-ray Scattering (MAXS and WAXS) studies.

The plot of the integrated intensity along the equator of a lying fiber of PBI **2** at 200 °C (Supplementary Figure 5) shows seven reflections indexed with the Miller indices to 100, 110, 200, 210, 300, 220 and 310 according to a Col_h lattice *p6mm* (a = 39.9 Å). In the WAXS pattern (Supplementary Figure 4 middle) the additional reflection at 4.1 Å is attributed to the π-π stacking distance. There are four meridional reflections at 13.8 Å, 6.9 Å, 3.7 Å and 3.6 Å, which can be best fitted to the layer lines *hk*7, *hk*14, *hk*26 and *hk*27 of a helix (Supplementary Figures 4 and 5). The reflection at layer line seven is attributed to the subunit axial translation of the PBIs, which is in agreement with a helical pitch of 96.6 Å. The quite strong reflection at 3.6 Å is in corresponds with the 0027 reflection caused by the helix.

For PBI **3** the reflection at layer line seven (14.1 Å) is attributed to the subunit axial translation. The quite strong reflection at 3.6 Å is in agreement with the 0028 reflection caused by the helix (Supplementary Figures 6-7).

For PBI **4** five reflections on the equator were indexed with Miller indices 100, 110, 200, 210 and 300 according to a Col_h phase (a = 32.3 Å) in the planar group p6mm (Supplementary Figure 8). Furthermore, at the meridian various intensities were obtained, from which only three at 7.1 Å, 6.9 Å and at 3.55 Å could be best attributed to the reflections 0016, 0018 and 0032, respectively. The intensity at 12.1 Å has its maximum between layer lines L = 9 and L = 10 and thus this intensity possesses an off-meridional position (Supplementary Figure 9). For PBI **4** two molecules can arrange in one columnar slice of a height of 14.2 Å at a density of 0.895 g cm^{-3} (224 °C) indicating a double-stranded helix. Since the first meridional reflection at the 16th layer line corresponds to d = 7.1 Å, a centered structure along the columnar axis is assumed, which results in the systematic absence of the meridional reflection 008. Therefore, the first sharp reflection that can be seen directly at the meridian corresponds to the half pitch. The helical pitch and repeat of the 8_1 helix was calculated to be 7.1 Å × 16 = 113.6 Å. The quite strong reflection at 3.55 Å is in agreement with the 0032 reflection caused by this helix.

Supplementary Table 2. Experimental and calculated d-spacings for PBIs **1-4**.

Compound	Phase	reflection	d_{obs} (Å)	d_{cal} (Å)	*hkl*	Lattice parameter
		1	28.1	28.0	010	
		2	23.2	23.0/23.3	110/1-10	
		3	20.5	20.5	200	a = 41.3 Å
		4	20.4	20.1	001	b = 28.2 Å
		5	18.9	19.0	10-1	c = 20.4 Å
		6	17.2	17.3	101	α = 96°
		7	16.5	16.4	210	β = 97°
	Cr	8	14.0	14.0	020	γ = 90°
**1**	(triclinic)	9	13.7	13.9	21-1	
	at 136 °C	10	13.1	13.1	2-1-1	δ =
		11	12.3	12.2	310	0.98 gcm^{-3}
		12	11.5	11.5	220	with four
		13	10.9	11.0	021	molecules in
		14	10.0	10.1	002	the unit cell
		15	9.4	9.5	102	
		16	9.1	9.1	130	
		17	8.9	9.0	4-1-1	
		1	39.6	39.4	110	
		2	35.0	34.9	200	
		3	24.0	23.9	020	
	LC	4	19.7	19.7	220	
**2**	Col_r (160 °C)	5	17.4	17.4	400	a = 69.7 Å
	c2mm	6	15.5	15.5	130	b = 47.8 Å
		7	13.4	13.4	510	
		8	12.0	12.0	040	
		9	4.8	4.8	halo	
		10	4.2	4.2	π-π	
		1	34.7	34.6	100	
		2	20.0	20.0	110	
		3	17.3	17.3	200	
	LC	4	13.1	13.1	210	
**2**	Col_h (200 °C)	5	11.5	11.5	300	a = 39.9 Å
	p6mm	6	10.0	10.0	220	
		7	9.6	9.6	310	
		8	4.7	4.7	Halo	
		9	4.1	4.1	π-π	
		1	35.5	35.4	100	
	LC	2	20.5	20.5	110	
**3**	Col_h (180 °C)	3	17.7	17.7	200	a = 40.2 Å
	p6mm	4	13.4	13.4	210	
		5	4.8	4.8	Halo	
		6	4.1	4.1	π-π	
		1	28.0	27.9	100	
		2	16.2	16.1	110	
	LC	3	14.0	14.0	200	
**4**	Col_h (224 °C)	4	10.6	10.6	210	a = 32.3 Å
	p6mm	5	9.3	9.3	300	
		6	5.6	5.5	Halo	
		7	4.3	4.3	π-π	

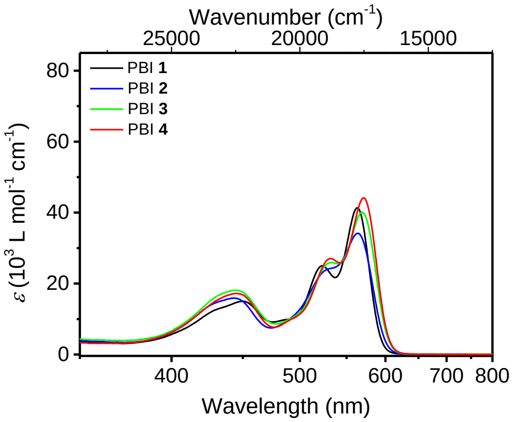

Supplementary Figure 11. UV-Vis absorption spectroscopy of PBIs 1-4. UV-Vis absorption spectra in dichloromethane at 25 °C (c = ~10^{-5} mol L^{-1}) of PBI **1** (black; λ_{max} = 562 nm), **2** (blue; λ_{max} = 562 nm), **3** (green; λ_{max} = 570 nm) and **4** (red, ; λ_{max} = 570 nm). Due to the high dilution and the good solvating properties of dichloromethane, no aggregates are formed and purely monomeric PBI dyes are present.

Supplementary Table 3. UV-Vis absorption properties of compounds **1**, **2**, **3** and **4.**

PBI*	0,0/0,1[a] (Monomer, DCM)	0,0/0,1[b] (Aggregate, solid state)
**1 (1)**	1.66	2.17
**2 (4)**	1.42	2.35
**3 (3)**	1.55	2.00
**4 (2)**	1.60	1.85

[a] CH_2Cl_2 (c = 1.2 - 1.9*10^{-5} M) at 25 °C; [b] drop-casted from $CHCl_3$ solution (c ~ 10^{-4} M) on quartz substrates. * numbers in brackets depict the respective numbers of strands.

Supplementary Note 2. UV-Vis investigations.

The change of the ratio of the first and second vibrational transition of the S_0-S_1 electronic transition of the aggregate was compared to the one of the monomer. Since the ratio increases in the solid state for aggregates, it shows that most of the oscillator strength is concentrated in the 0,0 transition, which is a typical signature for J-aggregates (Supplementary Figure 10 and Supplementary Table 2).

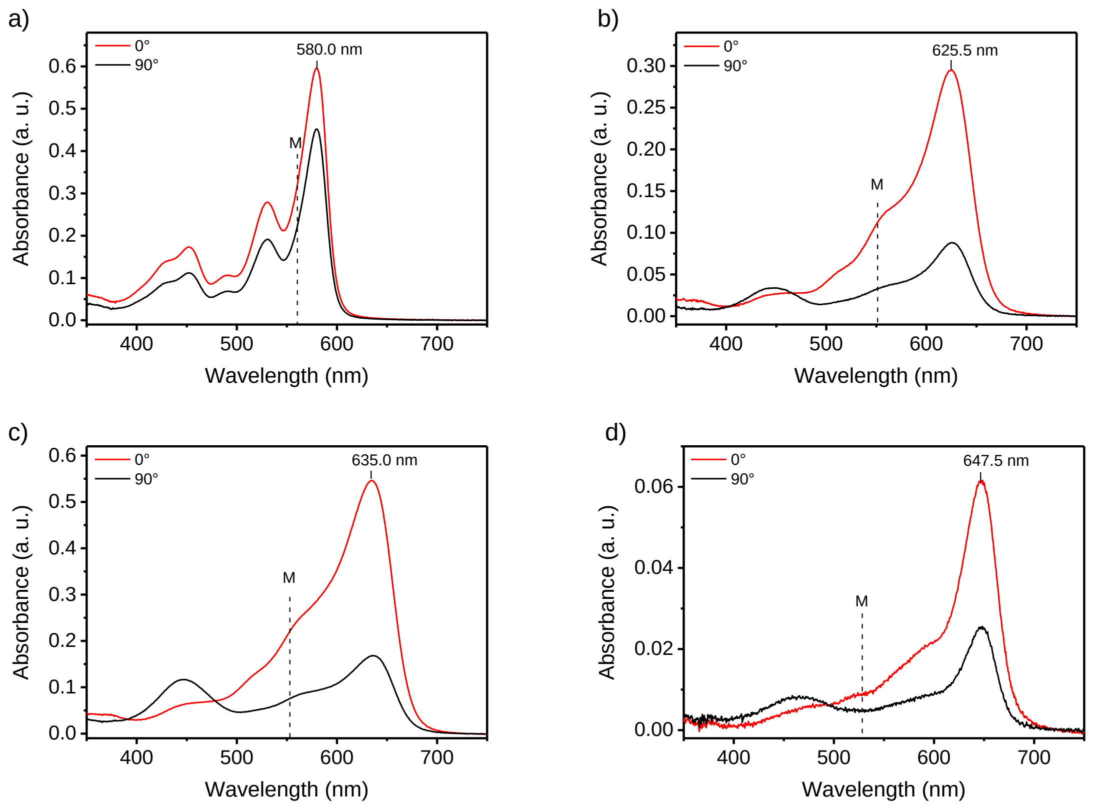

Supplementary Figure 12. Polarized UV-Vis absorption spectroscopy of PBIs 1-4. Polarized UV-Vis absorption spectra of thin films of PBI **1** (a), **2** (b), **3** (c) and **4** (d). Highest absorption intensity is observed when the incident light is parallel polarized to the shearing direction (red). Respective monomer absorption maxima in MCH are indicated by dashed lines (M).

Supplementary Note 3. Polarized UV-Vis measurements.

The change of the ratio of the first and second vibrational transition of the S_0-S_1 electronic transition of the aggregate was compared to the one of the monomer. Since the ratio increases in the solid state for aggregates, it shows that most of the oscillator strength is concentrated in the 0,0 transition, which is a typical signature for J-aggregates.

To describe the quality of the alignment the dichroic ratio D_λ and the order parameter S_λ were calculated for each PBI.[10]

$$S_\lambda = \frac{A_{\lambda,\max} - A_{\lambda,\min}}{A_{\lambda,\max} + 2A_{\lambda,\min}};$$ (Supplementary Equation 1)[10]

$$D_\lambda = \frac{A_{\lambda,\max}}{A_{\lambda,\min}}.$$ (Supplementary Equation 2)[10]

The respective calculated values for the dichroic ratio D_λ and the order parameter S_λ are as follows:

PBI **1:** $D_{580.0} = 1.31$, $S_{580.0} = 0.09$.

PBI **2:** $D_{625.5}$ = 3.33, $S_{625.5}$ = 0.44.

PBI **3:** D_{635} = 3.22, S_{635} = 0.43.

PBI **4:** $D_{647.5}$ = 2.44, $S_{647.5}$ = 0.32.

Because of the crystalline nature of PBI **1** a better alignment was not possible.

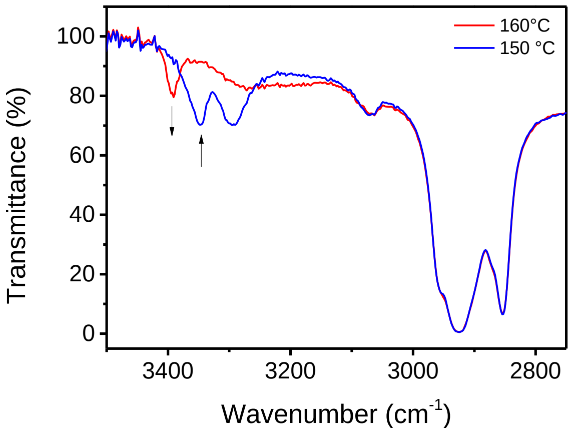

Supplementary Figure 13. Temperature-dependent FT-IR transmittance spectroscopy of PBI 1. Temperature-dependent FT-IR transmittance spectra of PBI **1** of the isotropic liquid phase at 160 °C and the crystalline phase at 150 °C. Arrows indicate the decrease of the free NH vibration band at 3390 cm^{-1} and the increase of the hydrogen-bonded NH vibration band at 3170 cm^{-1}.

Supplementary Note 4. FT-IR and polarized FT-IR experiments.

The PBI **1** sample was pressed between two KBr plates and heated up to 160 °C to form a thin film, which was investigated by temperature-dependent FT-IR measurements on a cooling process in transmission mode by using a heating stage. During decreasing temperature from 160 °C (isotropic liquid) to 150 °C (crystalline state) a new NH vibration band appeared at 3170 cm^{-1} while the NH vibration band at 3390 cm^{-1} completely disappeared (Supplementary Figure 12). According to literature, the NH vibration at 3390 cm^{-1} was assigned to free NH.[11-13] Considering the weakening of the NH bond strength upon H-bonding the raising of the NH vibration band at lower wavenumbers was assigned to the hydrogen-bonded NH between the imide groups in the crystalline state.

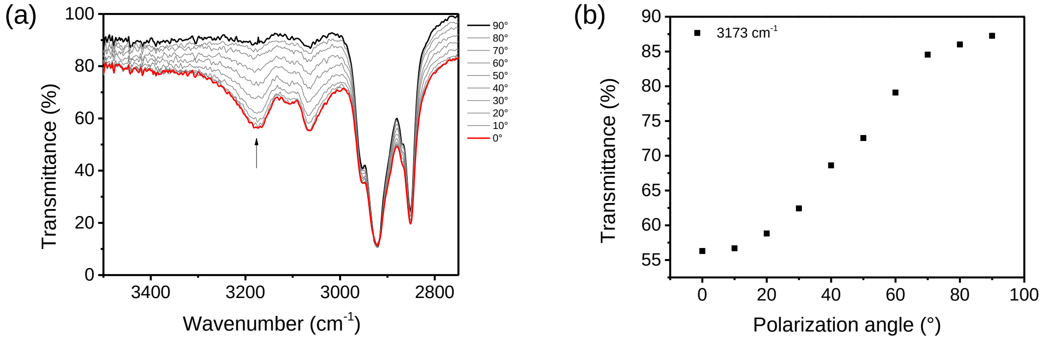

Supplementary Figure 14. Polarized FT-IR transmittance spectroscopy of PBI 2. (a) Polarized FT-IR transmittance spectra of an aligned thin film of PBI **2**. The spectra were measured with a polarizer oriented from 0° to 90° to the shearing direction. The arrow indicates the hydrogen bonded NH vibration at 3173 cm^{-1}. (b) Plot of FT-IR transmittance values at 3173 cm^{-1} of the aligned sample as function of the polarizer angle from 0° to 90°.

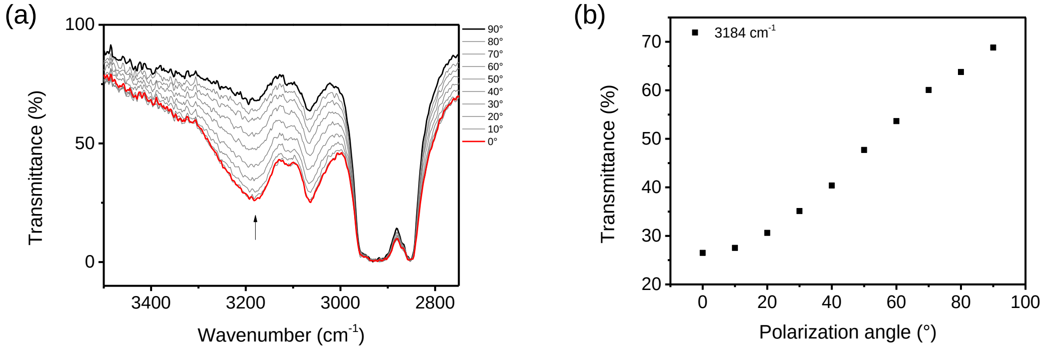

Supplementary Figure 15. Polarized FT-IR transmittance spectroscopy of PBI 3. (a) Polarized FT-IR transmittance spectra of an aligned thin film of PBI **3**. The spectra were measured with a polarizer oriented from 0° to 90° to the shearing direction. The arrow indicates the hydrogen bonded NH vibration at 3184 cm^{-1}. (b) Plot of FT-IR transmittance values at 3184 cm^{-1} of the aligned sample as function of the polarizer angle from 0° to 90°.

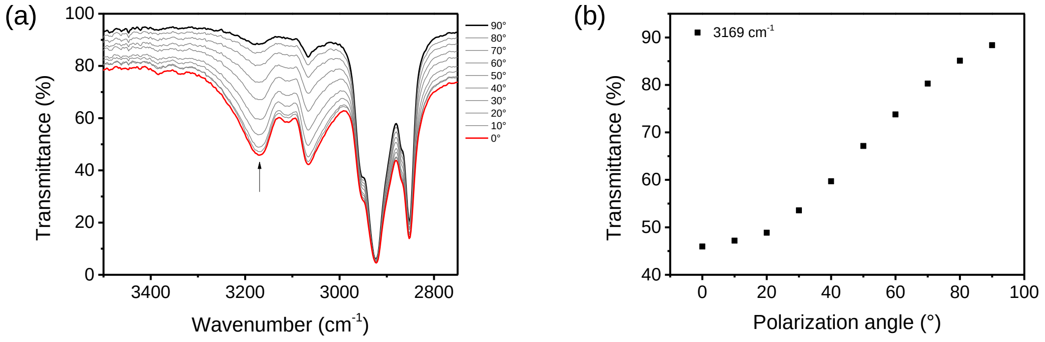

Supplementary Figure 16. Polarized FT-IR transmittance spectroscopy of PBI 4. (a) Polarized FT-IR transmittance spectra of an aligned thin film of PBI **4**. The spectra were measured with a polarizer oriented from 0° to 90° to the shearing direction. The arrow indicates the hydrogen bonded NH vibration at 3169 cm^{-1}. (b) Plot of FT-IR transmittance values at 3169 cm^{-1} of the aligned sample as function of the polarizer angle from 0° to 90°.

Supplementary Table 4. Calculated correlation lengths for π-π distances for PBIs **2**, **3** and **4**.

	PBI	ξ [Å]	N
m	**2** (Col_r)	15.46	3.7
	**2** (Col_h)	11.02	2.6
p	**3**	11.90	2.8
m,o	**4**	10.35	2.45

Supplementary Note 5. Calculation of the correlation lengths of π-stacks.

The correlation lengths ξ in the bulk state were calculated according to the Scherrer equation[14]:

$$\xi = (K \cdot \lambda)/(\Delta(2\theta) \cdot \cos(\theta)). \qquad \text{(Supplementary Equation 3)}$$

Where K is the dimensionless shape factor with a typical value of 0.9, λ the X-ray wavelength, $\Delta(2\theta)$ the line broadening at fwhm in radians and θ the Bragg angle in °.

$\Delta(2\theta)$ and θ were determined by fitting the peak on the equator belonging to the π-π stacking distance. The number of correlated molecules N was calculated referring to $N = \xi$ / π-π distance.

The calculated number of molecules, which are in strong correlation, supports the analysis of the self-assembled structures of double- (**4**), triple- (**3** and **2** at higher temperatures) and quadruple-stranded (**2** at lower temperatures).

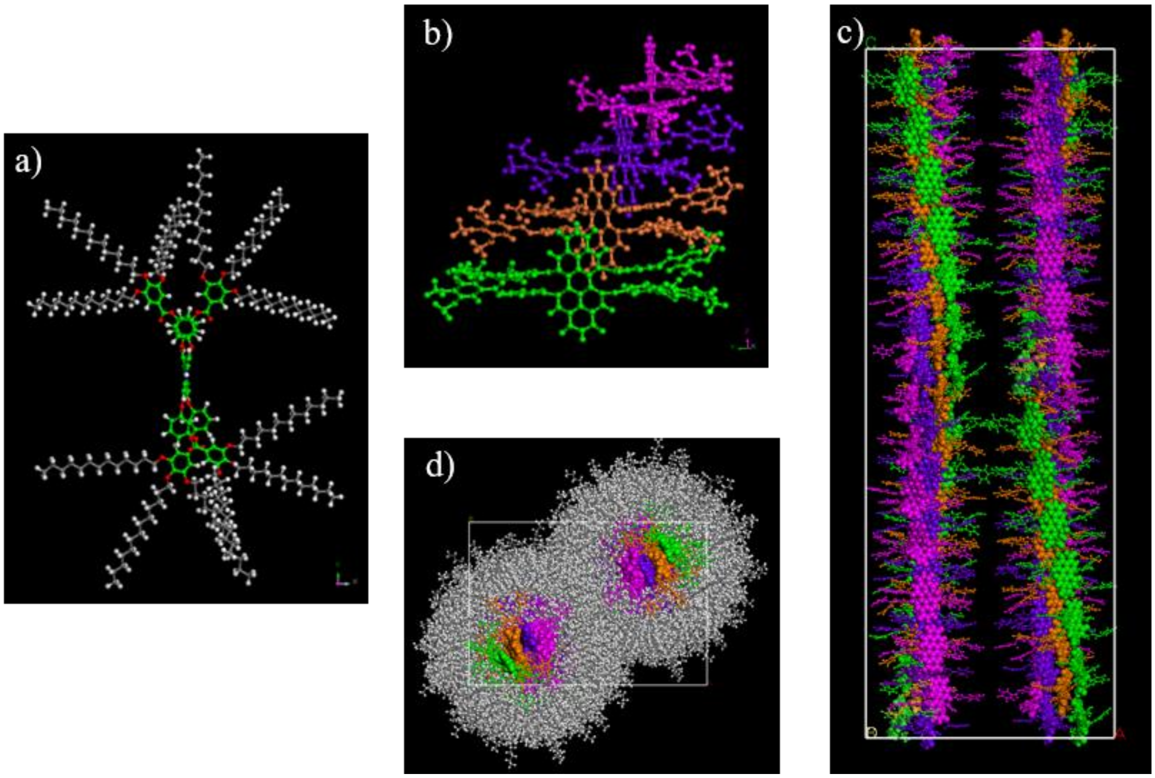

Supplementary Figure 17. Model of the self-assembly of PBI 2 at 160 °C. a) Geometry optimized single molecule in top view. b) Side view of the tetrameric unit without longer alkyl chains for more clarity. c) Side view of the unit cell, in which the quadruple strands are arranged body-centred. Aliphatic chains are omitted for clarity. The bay substituents are visualized in ball-stick and the PBI core in space filling representation. d) Top view of two columns with aliphatic chains of the geometry optimized structure. The PBI cores are colored in green, orange, blue and magenta to highlight the individual strands.

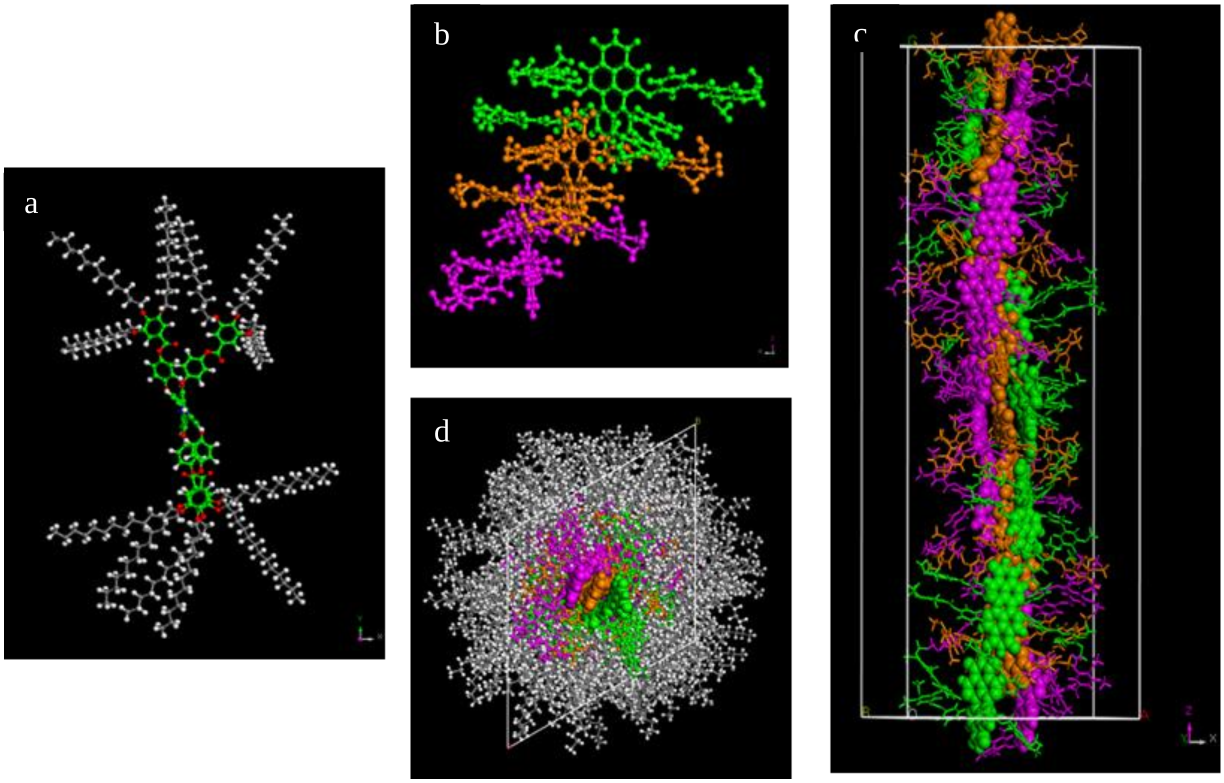

Supplementary Figure 18. Model of the self-assembly of PBI 3. a) Geometry optimized single molecule in top view. b) Side view of the trimeric unit without longer alkyl chains for more clarity. c) Side view of the unit cell with omitted aliphatic chains for clarity. The bay substituents are visualized in stick and the PBI core in space filling representation. d) Top view of one column with aliphatic chains of the geometry optimized structure. The PBI cores are colored in green, orange and magenta to highlight the individual strands.

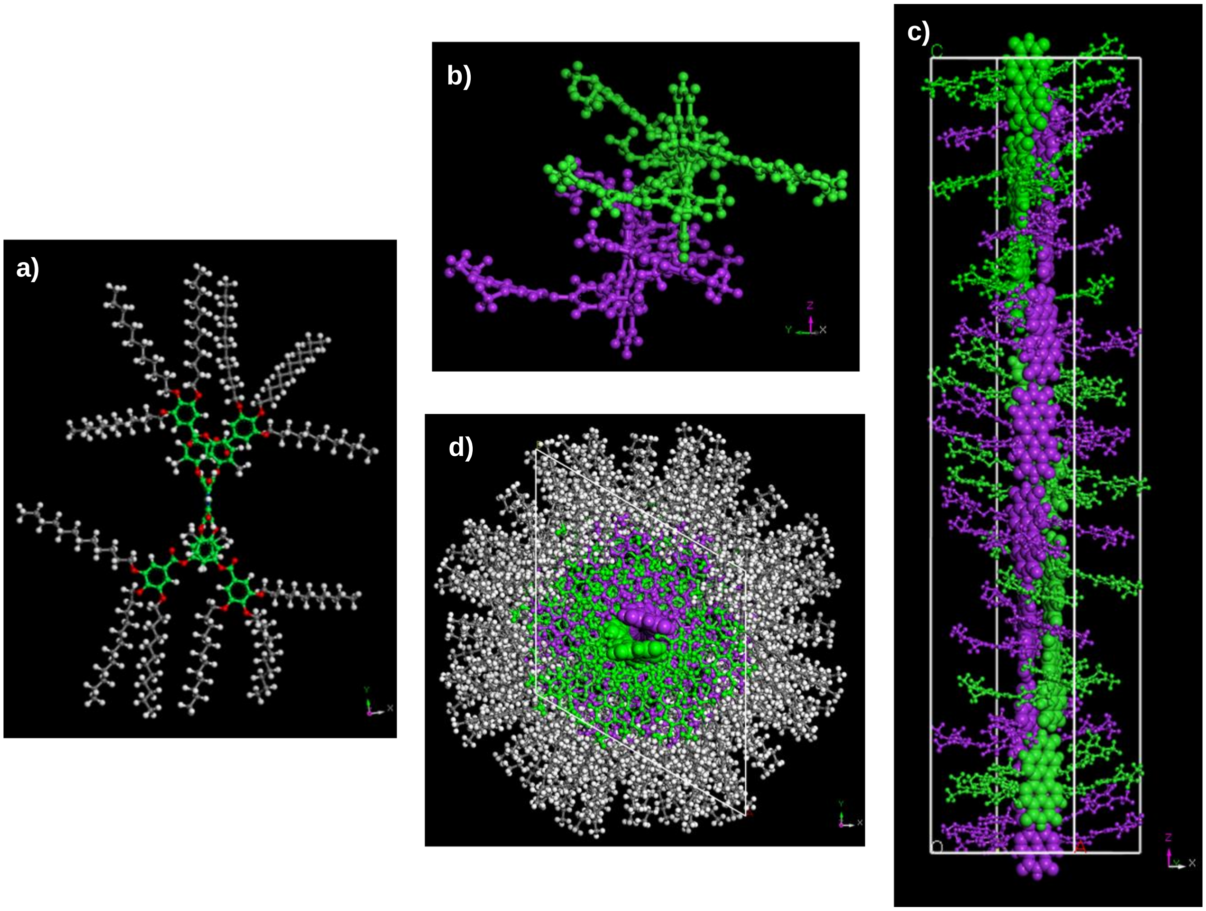

Supplementary Figure 19. Model of the self-assembly of PBI 4. a) Geometry optimized single molecule in top view. b) Side view of the dimer unit without longer alkyl chains for more clarity. c) Side view of one column of half size in the unit cell. Aliphatic chains are omitted for clarity. The bay substituents are visualized in ball-stick and the PBI core in space filling representation. d) Top view of one column with aliphatic chains of the geometry optimized structure. The PBI cores are colored in green and magenta to highlight the individual strands.

Supplementary Note 6. Modelling of the LC phases.

The assembly model of PBI **2** at 160 °C (Supplementary Figure 16), based on π-stacked tetramers, was generated by shifting each molecule by 7 Å and simultaneously rotate them to the prior one by 12.9°, which represents the intramolecular twist angle α, to prevent steric repulsion of the bulky substituents in bay position and to optimize the π-π stacking interactions. Those units were rotated by 2α = 25.8° with respect to each other. In sum 56 molecules were arranged in one helical column (14_1 helix). This gives helical strands of a length of 193.2 Å, in which each 14 PBI cores are connected by hydrogen bonding. Based on the density considerations and the *c2mm* symmetry of the columnar rectangular phase two columns composed of four strands and 112 mesogens each fill the total space of the unit cell (a = 69.7 Å, b = 47.8 Å) (Supplementary Figure 16c). This imposes that four strands must form a quadruple stranded helix by π-π interaction (quadruple-stranded 14_1 helix). This model has been geometry optimized by the force field COMPASS by an atom based summation method, until the non-bonding energy was strongly negative.

The assembly model of PBI **3** (Supplementary Figure 17), based on π-stacked trimers, was generated by shifting each molecule by 7 Å and simultaneously rotate them to the prior one by 25.7°, which represents the intramolecular twist angle α, to prevent steric repulsion of the bulky substituents in bay position and optimize the π-π stacking interactions. These trimer units were rotated by 2α = 51.4° with respect to each other. In sum 21 molecules were arranged in the Col_h unit cell (a = 40.2 Å) in one helical column (7_1 helix). This gives helical strands of a length of 96.6 Å, in which each 7 PBI cores are connected by hydrogen bonding. Based on the density considerations three strands fill the total space of the unit cell. This imposes that three strands must form a triple-stranded helix by π-π interaction (triple-stranded 7_1 helix) (Supplementary Figure 17c). This model has been geometry optimized by the force field COMPASS by an atom based summation method, until the non-bonding energy was strongly negative.

The assembly model of PBI **4** (Supplementary Figure 18), based on π-stacked dimers, was generated by shifting each molecule by 7 Å and simultaneously rotate them to the prior one by 22.5°, which represents the intramolecular twist angle α, to prevent steric repulsion of the bulky substituents in bay position and to optimize the π-π stacking interaction. In sum 16 molecules were arranged in the unit cell (a = 32.3 Å) in one helical column (8_1 helix). This gives helical strands of a length of 113.16 Å, in which each 8 PBI cores are connected by hydrogen bonding. Based on the density considerations two strands fill the total space of the unit cell. This imposes that two strands must form a double-stranded helix by π-π interaction (double-stranded 8_1 helix) (Supplementary Figure 18). This model has been geometry optimized by the force field COMPASS by an atom based summation method, until the non-bonding energy was strongly negative.

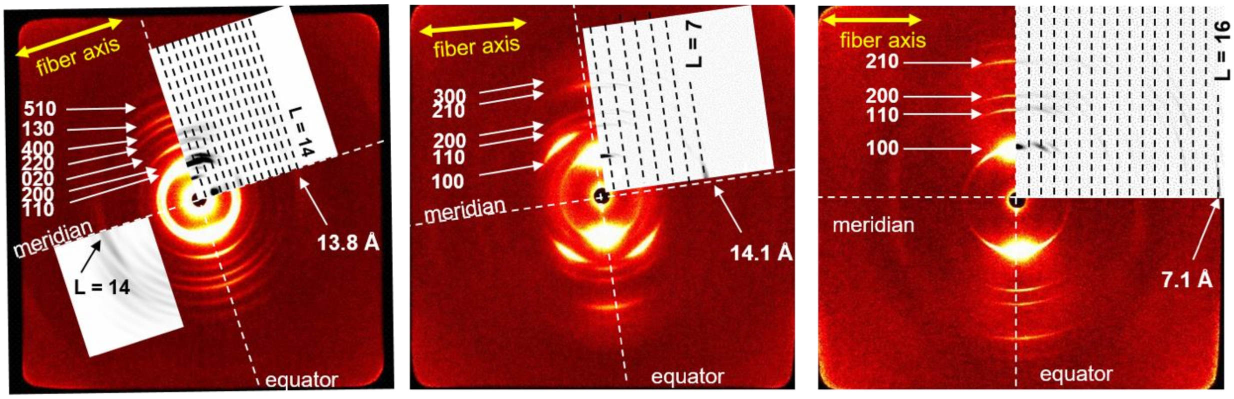

Supplementary Figure 20. MAXS patterns of PBIs 2-4. MAXS patterns of PBIs **2** at 160 °C (left), **3** at 180 °C (middle) and **4** at 224 °C (right), overlaid with the simulated diffraction patterns generated by CLEARER. White arrows indicate the accordance of the reflections on the equator and meridian.

Supplementary Note 7. Simulation of the fiber diffraction pattern.

The fiber diffraction patterns of PBI **2** (Col_r), **3** and **4** were simulated with CLEARER using the modelled structure, which were obtained via *Materials Studio*. The data were exported as PDB-files and loaded into the Fiber Diffraction Simulation module of CLEARER.[15] To fit the pattern the fiber axis was set to (0,0,1) with a "crystallite size" of 130 nm (*a*), 130 nm (*b*) and 60 nm (*c*). The "crystal size" has been adapted to best fit the pattern. The cell dimensions were set to experimental parameters. The fiber disorder parameters σ_θ and σ_ϕ were 0.08 radians and infinite with a sample interval of 1 pixel. The contrast was adjusted to best visualize the signals of the pattern. It has to be considered that in this program the liquid crystal fiber is simulated as a perfectly ordered domain of a given size on the basis of one unit cell with the given orientational disorder of the domains. In comparison, a real liquid crystal is not built with identical unit cells. In addition, the model cannot be simulated simultaneously with conveniently ordered aromatic building blocks and liquid-like chains, since the geometry optimization optimizes always both building blocks. For the most complex rectangular unit cell the geometry optimization affords a unit cell deviating from the body-centred structure and therefore also reflections may reveal, which are extinct in the experimental pattern. These factors have impact on all intensities of the diffraction patterns. However, the most important features of the experimental diffraction results are qualitatively well reproduced by the simulation.[16]

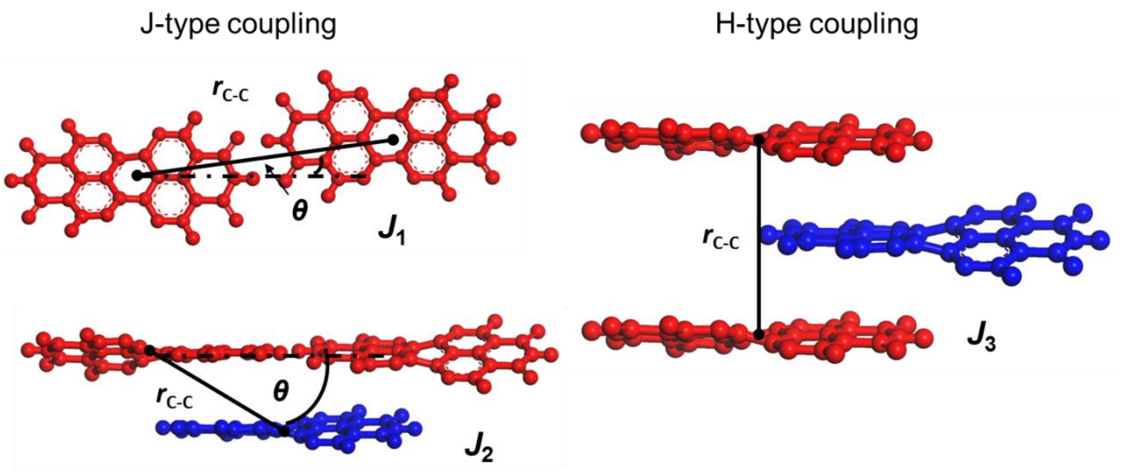

Supplementary Figure 21. Exciton coupling between PBI chromophores. Schematic illustration of the self-assemblies in slipped J-type strands and the distances and angles used for the calculations for the J- and H-type coupling.

Supplementary Table 5. Exciton coupling energies J calculated for neighboring molecules of PBI **1**, **2**, **3** and **4** in H-bond (J_1), slipped π-π stack (J_2) and side-by-side π-π stack direction (J_3).[a]

	PBI		μ_{eg} [D]	$r_{c\text{-}c}$ [Å]	θ [°]	J_i [cm^{-1}]
		J_1		14.20	0	−156
o	**1**	J_2	6.6	-	-	-
		J_3		-	-	-
		J_1		14.10	5.2	−155
m	**2**	J_2	6.6	7.80	28.4	−611
		J_3		8.40	90	+370
		J_1		14.10	9.9	−154
p	**3**	J_2	6.7	7.80	28.1	−636
		J_3		8.20	90	+435
		J_1		14.20	7.7	−173
o,m	**4**	J_2	7.1	7.95	31.5	−597
		J_3		-	90	-

[a] Exciton coupling energies J were calculated within the point-dipole approximation following Kasha's theory. The sign (+/−) specifies the type of contribution and is negative exciton coupling energy (J-type coupling) and positive exciton coupling energy (H-type coupling). α is always 0°.

Supplementary Note 8. Exciton Theory.

Distances (r) and angles (α, θ) were measured with the *Materials Studio* program from the center of one PBI to the center of the adjacent PBI molecule in π-π stack and H-bond direction. The distances for the calculations of H-type couplings were directly taken from the analysis of the X-ray data.

We use exciton theory according to Kasha[17] with the PDA to explain the bathochromic shifts of the absorption bands of the aggregates and elucidate the differences in the spectral shifts that originate from the aggregate structures of the one-, two-, three- and four-stranded helices.

The transition dipole moment was calculated from the integral of the reduced UV-Vis absorption band according to:

$$\left|\mu_{eg}\right|^2 = \frac{3hc\varepsilon_0 \ln 10}{2\pi^2 N_A} \cdot \int_{\widetilde{\nu_1}}^{\widetilde{\nu_2}} \frac{\varepsilon(\tilde{\nu})}{\tilde{\nu}} \, d\tilde{\nu} \qquad \text{(Supplementary Equation 4)}$$

Where $\varepsilon(\tilde{\nu})$ is the molar extinction coefficient, c is the speed of light, h is the Planck constant and N_A is the Avogadro constant.

Besides the transition dipole moment distances as well as the slip angles are of importance for the respective coupling as it can be seen from the equation[17]:

$$J_i = \frac{\left|\mu_{\text{eg}}\right|^2}{4\pi\varepsilon_0 r_{\text{uv}}^3} \left(1 - 3\cos^2\theta\right) \qquad \text{(Supplementary Equation 5)}$$

Where μ_{eg} is the transition dipole moment of the monomer, ε_0 is the permittivity in vacuum, r_{uv} is the distance between neighboring molecule centers and θ is the slip angle resulting from the translational offset of two parallel arranged molecules.

Due to the simplicity of Kasha's exciton theory the derived values are too large and confirm the limitations of the PDA for close intermolecular distances. Because of the very large molecular structures further quantum mechanical investigations are, however, not possible.

Supplementary References

1 Kaiser, T. E., Stepanenko, V. & Würthner, F. Fluorescent J-Aggregates of Core-Substituted Perylene Bisimides: Studies on Structure-Property Relationship, Nucleation-Elongation Mechanism, and Sergeants-and-Soldiers Principle. *J. Am. Chem. Soc.* **131**, 6719-6732 (2009)

2 Herbst, S., Soberats, B., Leowanawat, P., Lehmann, M. & Würthner, F. A Columnar Liquid Crystal Phase Formed by Hydrogen-Bonded Perylene Bisimide J-Aggregates. *Angew. Chem. Int. Ed.* **56**, 2162-2165 (2017).

3 Kaiser, T. E., Wang, H., Stepanenko, V. & Würthner, F. Supramolecular construction of fluorescent J-aggregates based on hydrogen-bonded perylene dyes. *Angew. Chem. Int. Ed.* **46**, 5541-5544 (2007).

4 Moore, J. S. & Stupp, S. I. Room temperature polyesterification. *Macromolecules* **23**, 65-70 (1990).

5 Mao, S., Duan, Z., The P, V, T, x properties of binary aqueous chloride solutions up to T = 573 K and 100 MPa, *J. Chem. Thermodyn*. **40**, 1046-1063 (2008).

6 Lehmann, M., Jahr, M., Donnio, B., Graf, R., Gemming, S., Popov, I., Star-Shaped Oligobenzoates: Non-conventional Mesogens Forming Columnar Helical Mesophases, *Chem. Eur. J.* **14**, 3562-3576 (2008).

7 Xiao, Y. *et al.* Chemical engineering of donor–acceptor liquid crystalline dyads and triads for the controlled nanostructuration of organic semiconductors, *Cryst. Eng. Comm.* **18**, 4787–4798 (2016).

8 Donnio, B., *et al.* A Generalized Model for the Molecular Arrangement in the Columnar Mesophases of Polycatenar Mesogens. Crystal and Molecular Structure of Two Hexacatenar Mesogens, *J. Am. Chem. Soc.* **126**, 15258 – 15268 (2004).

9 Uhríková, D., Rybár, P., Hianik, T., Balgavý, P., Component volumes of unsaturated phosphatidylcholines in fluid bilayers: a densitometric study, *Chem. Phys. Lipids* **145**, 97–105 (2007).

10 Gsänger, M. *et al.* High-Performance Organic Thin-Film Transistors of J-Stacked Squaraine Dyes. *J. Am. Chem. Soc.* **136**, 2351-2362 (2014).

11 van der Weegen, R. *et al.* Small sized perylene-bisimide assemblies controlled by both cooperative and anti-cooperative assembly processes. *Chem. Commun.* **49**, 5532-5534 (2013).

12 Zou, S. et al. Tunable Mesogens Based on Shape-Persistent Aromatic Oligoamides: From Lamellar, Columnar, to Nematic Liquid Crystalline Phase. *Org. Lett.* **14**, 3584-3587 (2012).

13 *Spectroscopic Methods in Organic Chemistry*; Hesse, M., Meier, H., Zeeh, B., ed. 2nd; Thieme: Stuttgart, (2007).

14 a) *Chemical Analysis: Introduction to X-ray Powder Diffractometry*. Jenkens, R., Snyder, R. L., Vol. 138, Wiley, New York, (1996). b) Scherrer, P. Bestimmung der Grösse und der Inneren Struktur von Kolloidteilchen Mittels Röntgenstrahlen, Nachrichten von der Gesellschaft der Wissenschaften. *Nachr. Ges. Wiss. Göttingen, Math.-Phys. Kl.*, **2**, 98-100 (1918).

15 Makin, O. S., Sikorski, P. & Serpell, L. C. CLEARER: a new tool for the analysis of X-ray fibre diffraction patterns and diffraction simulation from atomic structural models. *J. Appl. Crystallogr.* **40**, 966-972 (2007).

16 Since the authors of the CLEARER program state that the program is limited to small unit cells of up to maximum 60 Å, we approved the results also with the module Powder Diffraction in the program suite “Materials Studio”.

17 Kasha, M. & Rawls, H. R., El-Bayoumi, M. A. The exciton model in molecular spectroscopy. *Pure Appl. Chem.* **11**, 371–392 (1965).
